# Health Literacy Influences Heart Failure Knowledge Attainment but Not Self-Efficacy for Self-Care or Adherence to Self-Care over Time

**DOI:** 10.1155/2013/353290

**Published:** 2013-07-24

**Authors:** Aleda M. H. Chen, Karen S. Yehle, Nancy M. Albert, Kenneth F. Ferraro, Holly L. Mason, Matthew M. Murawski, Kimberly S. Plake

**Affiliations:** ^1^Assistant Professor of Pharmacy Practice, School of Pharmacy, Cedarville University, 251 N. Main Street, Cedarville, OH 45314, USA; ^2^School of Nursing, Purdue University, 502 N. University Street, JNSN 238, West Lafayette, IN 47907, USA; ^3^Center on Aging and the Life Course, Purdue University, West Lafayette, IN 47907, USA; ^4^Regenstrief Center for Healthcare Engineering, Purdue University, West Lafayette, IN 47907, USA; ^5^Office of Research & Innovation, Nursing Institute and CNS, Kaufman Center for Heart Failure, Heart and Vascular Institute, Cleveland Clinic, 9500 Euclid Avenue, J3-4, Cleveland, OH 44195, USA; ^6^Distinguished Professor of Sociology, Purdue University, Bill and Sally Hanley Hall, 1202 W. State Street, West Lafayette, IN 47907, USA; ^7^Pharmacy Administration, College of Pharmacy, Purdue University, Heine Pharmacy Building, 575 Stadium Mall Drive, West Lafayette, IN 47907, USA; ^8^Pharmacy Practice, College of Pharmacy, Purdue University, Heine Pharmacy Building, 575 Stadium Mall Drive, West Lafayette, IN 47907, USA

## Abstract

*Background*. Inadequate health literacy may be a barrier to gaining knowledge about heart failure (HF) self-care expectations, strengthening self-efficacy for self-care behaviors, and adhering to self-care behaviors over time. *Objective*. To examine if health literacy is associated with HF knowledge, self-efficacy, and self-care adherence longitudinally. *Methods*. Prior to education, newly referred patients at three HF clinics (*N* = 51, age: 64.7 ± 13.0 years) completed assessments of health literacy, HF knowledge, self-efficacy, and adherence to self-care at baseline, 2, and 4 months. Repeated measures analysis of variance with Bonferroni-adjusted alpha levels was used to test longitudinal outcomes. *Results*. Health literacy was associated with HF knowledge longitudinally (*P* < 0.001) but was not associated with self-efficacy self-care adherence. In posthoc analyses, participants with inadequate health literacy had less HF knowledge than participants with adequate (*P* < 0.001) but not marginal (*P* = 0.073) health literacy. *Conclusions*. Adequate health literacy was associated with greater HF knowledge but not self-efficacy or adherence to self-care expectations over time. If nurses understand patients' health literacy level, they may educate patients using methods that promote understanding of concepts. Since interventions that promote self-efficacy and adherence to self-care were not associated with health literacy level, new approaches must be examined.

## 1. Introduction

Heart failure is identified as a leading cause of hospitalizations [1], morbidity, mortality, and rising healthcare costs for nearly six million Americans [2, 3]. After a diagnosis of heart failure, patients must perform self-care behaviors to reduce negative clinical outcomes [4, 5]. Self-care is a decision-making process, where patients perform activities to prevent symptoms (maintenance) and respond to symptoms as they occur (management) [4]. Self-care maintenance activities for heart failure patients include exercising daily, eating a low sodium diet, monitoring fluid intake, and monitoring weight. Patients may respond to symptoms by engaging in the following self-care management activities: consulting their healthcare provider, reducing fluid and sodium intake, and increasing the dose of a diuretic. However, patients' adherence to recommended self-care behaviors varies greatly and is generally poor [5–7].

Multiple factors may affect patients' adherence to heart failure self-care including heart failure knowledge. Patients may not have received recommended heart failure education [8, 9] if the heart failure diagnosis was secondary to another health problem, such as myocardial infarction, resulting in inadequate knowledge about heart failure [10]. Initial education about heart failure often occurs during hospitalization when the patient may be too ill or overwhelmed with acute care events, potentially reducing retention of information presented unless family members are available to be counseled [10]. Additional education occurs in the outpatient setting, but content variability can affect overall heart failure knowledge. Further, chronic heart failure is a complex condition to self-management. Patients must monitor their sodium intake, manage medications, manage fluids, perform physical activity, assess signs and symptoms of worsening condition, and follow up with healthcare providers [5, 8, 9]. Adherence to heart failure self-care regimens requires that patients apply heart failure knowledge and education principles when making decisions and managing situations [9]. Even when patients receive additional heart failure and self-care education in an outpatient setting based on clinical practice guidelines [8, 10], inadequate health literacy is a potential barrier that prevents knowledge and skills acquisition [5, 11–13].

Health literacy, defined as obtaining, understanding, and using health information, may impact knowledge gained during heart failure education and patient adherence to self-care in heart failure [13]. Prevalence of inadequate health literacy in patients with heart failure ranges from 17.5 to 41% [11, 14–16]. There is no consensus regarding the impact of health literacy on heart failure outcomes [13]. Patients with inadequate health literacy had less heart failure knowledge [17–19] and less adherence to heart failure related self-care regimen expectations [20, 21]; however, in a similarly designed, cross-sectional study, other researchers found no relationship between health literacy and self-care adherence [18]. 

Self-efficacy also may be influenced by health literacy. Self-efficacy, derived from Bandura's social cognitive theory, is defined as an individual's confidence in his or her ability to perform health behaviors [22, 23]. The level of self-efficacy an individual possesses influences adherence to goals and responses to challenges [22, 23]. Lack of disease-specific knowledge due to inadequate health literacy also may affect patients' self-efficacy regarding their ability to adhere to complex self-care regimens. If individuals lacked self-efficacy (i.e., confidence) regarding their decisions, they did not carry out appropriate self-care [18, 19]; however, in an other research, a lack of patient self-efficacy did not alter adherence to self-care regimens [20].

Educational interventions designed for patients with inadequate health literacy are thought to improve disease knowledge and self-care adherence. Although educational interventions for patients with heart failure and inadequate health literacy improved knowledge, self-efficacy, daily weight measurements [11], and medication adherence [15, 21], one group of researchers found that the effects of education did not last past the intervention [15]. Previously, much of the research on health literacy in heart failure was focused on the impact of inadequate health literacy. For different health literacy levels, little is known about their association with changes in heart failure knowledge, self-efficacy for self-care, and adherence to self-care over time. 

## 2. Objectives

The objectives of this study were to examine associations between health literacy level (inadequate, marginal, and adequate) and heart failure knowledge, self-efficacy for self-care, and self-care adherence longitudinally over a four-month period in community-dwelling adults.

## 3. Methods

This multicenter study used a correlational, longitudinal design with three data collection periods; baseline, two, and four months. Institutional Review Board (IRB) approval was obtained from each clinical data collection site and Purdue University.

### 3.1. Participants and Procedures

Participants were recruited from 2009 to 2011 at three heart failure clinics: Cleveland Clinic in the Heart and Vascular Institute (Cleveland, OH, USA), Indiana University Health-Bloomington Hospital HEARTTEAM Cardiopulmonary Rehab and Congestive Heart Failure Center (Bloomington, IN, USA), and Community Health Network Indiana Heart Hospital Healthy Hearts Center (Indianapolis, IN, USA). At each site, heart failure patient education was provided as part of standard care procedures and typically completed in the first two months of care. Education in these clinics is provided primarily by advanced practice nurses (APNs) or registered nurses with consults from registered dieticians or other healthcare providers as applicable. Content is based on heart failure guidelines and includes heart failure diagnosis, self-care, medications, diet, and exercise. The settings differed in that the environments of care were urban, rural, and community based, respectively. 

Nursing staff identified new clinic referrals who would meet study inclusion criteria, a new clinic referral, at least 18 years of age, able to read and speak English, and no cognitive impairment based on clinical judgment. Patients were excluded if they resided in a skilled nursing facility or received home healthcare services. Eligible adult patients with heart failure were invited to participate at the initial clinic appointment by researchers who were not involved in direct patient care. 

Questionnaires were administered by trained researchers or research assistants; direct patient care providers were not involved in recruitment or data collection. At baseline, questionnaires were administered in private areas of each outpatient heart failure clinic before patients received education. At two, and four months, questionnaires were mailed to participants from the Bloomington Clinic and Community Health Network and were completed via telephone or by mail (at participant's request) at the Cleveland Clinic. The two-months data collection point was chosen as patients completed education by two months. This allowed for a two months period without scheduled education before the four-month assessment. 

### 3.2. Measures

 Health literacy was measured using the *Short-Form Test of Functional Health Literacy* (S-TOFHLA) [24]. The S-TOFHLA consists of 36 reading comprehension items, which contain examples of commonly used healthcare materials, and is required to be completed within a 7-minute time frame. Scores were categorized as recommended: inadequate (0–16 points), marginal (17–22 points), and adequate (23-36 points). The S-TOFHLA is a reliable and valid measure of health literacy, with Cronbach's alpha of 0.98 and established criterion validity [24]. 

Knowledge of heart failure was measured using the *Heart Failure Knowledge Questionnaire *(HFKQ). The HFKQ contains 14 close-ended items and one open-ended, item, and content includes heart failure pathology, symptoms, medications, and self-management. Scores range from 0 (lack of knowledge) to 15 (knowledgeable) and the previously reported Cronbach's alpha of 0.62 [6]. In this study, Cronbach's alpha at baseline assessment (*n* = 81) was similar at 0.66. 

Self-efficacy for heart failure self-care and adherence to heart failure self-care behaviors were measured using the *Self-Care Heart Failure Index* v.6 (SCHFI) that assesses adherence to both self-care maintenance and management behaviors [4, 25]. Of 22 items, 6 items measure self-efficacy, 10 items measure self-care maintenance, and 6 items measure self-care management. Items were rated on a four-point response scale from 1 = never or rarely to 4 = always or daily for the maintenance subscale, from 1 = not confident to 4 = extremely confident for the confidence subscale, and 1 = not quickly, not likely, or not sure to 1 = very quickly, very likely, very sure for the management subscale; then each subscale score was standardized to 100 points [25]. In order to score subscale B (self-care management), patients must have experienced an exacerbation of heart failure within the past two months. A score of ≥70 was used as the cut-point to reflect self-care adequacy in each subscale. Psychometric performance of SCHFI was assessed previously and found to be valid and reliable (maintenance: alpha = 0.553, management: alpha = 0.597, confidence/self-efficacy: alpha = 0.827, and combined maintenance/management: alpha = 0.798) [4, 25, 26]. 

Patient characteristics were obtained at baseline and included gender, age, marital status, ethnicity/race, education, income, body mass index (BMI), and number of prescription medications. 

### 3.3. Data Analysis

Descriptive statistics were calculated for patient characteristics and included frequencies and percentages for categorical variables and means and standard deviations for continuous variables. Associations between patient characteristics (age, education, BMI, and prescription medications) and study outcomes (heart failure knowledge, self-efficacy for self-care, and self-care adherence) were examined using Pearson correlations. Difference in baseline patient characteristics and characteristics of patients who completed all follow-up evaluations were assessed using *t*-tests, Mann-Whitney tests, or One-Way Analysis of Variance (ANOVA) with Bonferroni corrections for multiple comparisons, as appropriate. Differences in characteristics of patients who completed all follow-up evaluations by health literacy level were assessed using *t*-, Chi-squared, or Kruskal-Wallis tests, as appropriate. Differences in characteristics of patients who completed all follow-up evaluations by study outcome (heart failure knowledge, self-efficacy for self-care, self-care maintenance, and self-care management) were assessed using Pearson correlations or One-Way Analysis of Variance (ANOVA) with Bonferroni corrections for multiple comparisons, as appropriate.

Means and standard deviations were calculated for health literacy at baseline and for knowledge, self-efficacy, and self-care at each assessment period. A power analysis was performed to with a power of 0.8, an alpha of 0.05, and a medium effect size. From that power analysis, a sample size of at least 36 participants was needed to perform the repeated measures ANOVA. Repeated measures ANOVA were performed to determine if differences existed over time, and when significant differences were found, Bonferroni corrections were used to perform multiple comparisons. Profile plots also were generated. An *a priori* level of 0.05 was used for statistical significance. All analyses were performed using IBM SPSS v. 19.0 for Windows (Armonk, NY, USA).

## 4. Results

### 4.1. Participant Characteristics

Eighty one participants completed baseline questionnaires; however, analyses were based on participants (*n* = 51) who completed two-month and/or four-month assessments. Participants were generally young compared to registry data on heart failure, white, graduated from high school, and took nearly 9 prescription medications on a regular basis. Compared to the 81 patients who enrolled in the study, those completing follow-up data collections (*n* = 51) were not significantly different (*P* > 0.05, data not shown). All results hereafter will include only patients who completed all follow-up data collections (*N* = 51). There were significant differences by age, BMI, recruitment site, and marital status by health literacy level (Table 1). Participants with inadequate health literacy were significantly older and were more likely to be recruited from the Bloomington Hospital site. Participants with marginal health literacy had significantly higher BMI than those with adequate health literacy.

Of participant characteristics, there were differences in heart failure knowledge by age, years of education, recruitment site, and marital status (Table 2). In Bonferroni-adjusted posthoc tests for recruitment site and marital status, participants at Cleveland Clinic had significantly more knowledge at baseline than Bloomington Hospital (*P* = 0.015) and CHN (*P* = 0.029). Participants who were married had significantly more knowledge than those who were widowed at baseline (*P* = 0.001), two (*P* = 0.002), and four months (*P* = 0.004).

There were differences in self-efficacy for self-care by recruitment site and BMI. In Bonferroni-adjusted posthoc tests, participants at Bloomington Hospital had significantly lower self-efficacy for self-care than participants at the CHN site (*P* = 0.032) at baseline. BMI was negatively associated with self-efficacy for self-care at baseline and two months. 

There were differences in self-care maintenance by recruitment site. In Bonferroni-adjusted posthoc tests, participants at Cleveland Clinic had significantly higher self-care maintenance adherence than participants at the CHN site (*P* = 0.026) at 4 months. There were no other significant differences in or associations with outcomes based on participant characteristics.

### 4.2. Adequacy of Health Literacy and Outcomes

At baseline, mean health literacy was adequate, but heart failure knowledge was low (failing mean score by testing standards), and self-efficacy for self-care and adherence to self-care maintenance and management behaviors were below cut off scores, reflecting inadequacy (Table 3). Of participants, 41.2% had adequate self-efficacy for performing self-care at baseline.

By the four-month followup, knowledge level remained low but increased to 64% (equaling a “D grade” by testing standards), and self-efficacy for self-care behaviors and adherence to self-care increased to adequate levels. Patient knowledge and self-care maintenance significantly improved over time (*P* = 0.012 and *P* = 0.002, resp.), but patient self-care management and self-efficacy did not significantly improve over time (*P* = 0.754 and *P* = 0.148, resp.). 

### 4.3. Assessment of the Impact of Baseline Health Literacy over Time

**Figure 1:**
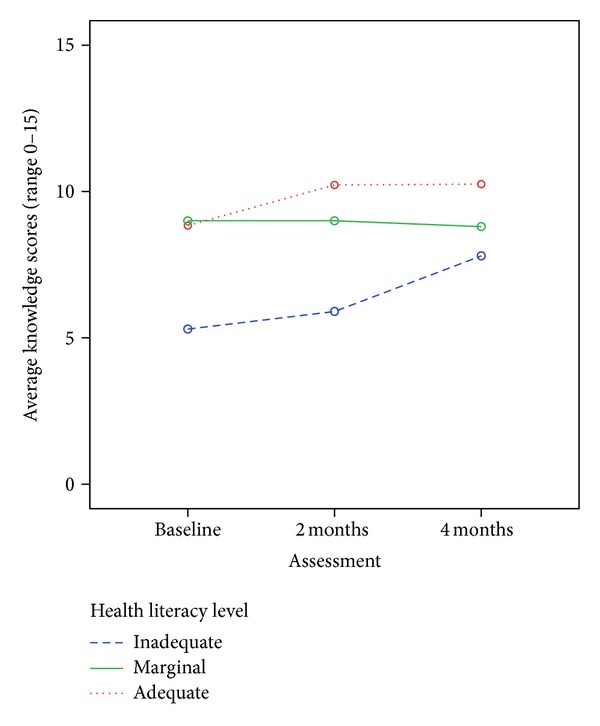
Changes in knowledge by health literacy level over time.

Health literacy categories at baseline were used to assess outcomes over time (Table 4). There were significant effects of health literacy on heart failure knowledge over time, but no effects of health literacy on other outcomes (self-efficacy and self-care). There was a significant effect of time on heart failure knowledge. There was no time-health literacy interaction, as evidenced by a nonsignificant *P* value and the profile plot (Figure 1), which indicated significant effects of both time and health literacy.

To further examine the differences in knowledge by health literacy level, Bonferonni-adjusted posthoc tests were performed, and patients with inadequate health literacy had significantly less knowledge than those with adequate (*P* < 0.001) but not marginal (*P* = 0.073) health literacy, as seen in Figure 1. Although patients with inadequate health literacy had a larger rise in heart failure knowledge score at 4 months compared to those with marginal and adequate health literacy at baseline, heart failure knowledge levels remained below that of patients with adequate health literacy (Figure 1).

## 5. Discussion

In this study, the importance of health literacy on heart failure knowledge score, self-efficacy for heart failure self-care, and adherence to heart failure self-care was examined over a four-month period. There were positive, longitudinal associations between health literacy and knowledge (higher health literacy with greater knowledge) but not between health literacy and self-efficacy for self-care or self-care adherence. Traditional clinic-based education improved knowledge overall, but the knowledge level of individuals with inadequate health literacy never improved to the level of those with adequate health literacy. Therefore, traditional clinic-based education may not be the best method to improve heart failure knowledge gaps over time for patients with inadequate health literacy. Moreover, since adherence to heart failure self-care behaviors improves clinical outcomes in heart failure [5, 11, 27], determining reasons for nonadherence, beyond health literacy, may be a key element in promoting heart failure self-care maintenance and management.

Disease-specific education has been found to improve knowledge in heart failure [11, 28, 29]. In this study, patients with inadequate and adequate health literacy experienced gains in knowledge during traditional clinic-based education. DeWalt and colleagues found education for patients with inadequate health literacy improved heart failure knowledge [11]. Similarly, we found that patients with inadequate health literacy demonstrated improved heart failure knowledge over the course of traditional clinic-based education. Over time, patients with inadequate health literacy continued to experience knowledge gains but had less heart failure knowledge than patients with adequate health literacy across both assessments. Since the distribution of inadequate literacy patients in this study mirrors other research and the health literacy levels are representative of the general heart failure population [11, 14, 15], the results of this study indicate that traditional education efforts may not reduce the knowledge disparity between patients with inadequate and adequate health literacy. Furthermore, researchers found in a diabetes educational intervention that although all patients gained considerable knowledge, patients with low health literacy did not gain as much as higher health-literate patients [30]. 

In three other studies, researchers consistently found that health literacy and patient heart failure knowledge are related [17–19]. Similar to our study, these studies used the TOFHLA [19] or the S-TOFHLA [17, 18] to measure health literacy, but each study utilized different measures of heart failure knowledge. Despite differences in measuring heart failure knowledge, other studies confirmed our findings that patients with inadequate health literacy had less heart failure knowledge. Furthermore, posthoc power analyses revealed that there was sufficient power to examine the difference (using repeated measures ANOVA) between health literacy categories with regard to knowledge (partial *η* ^2^ = 0.316, power = 0.988). Clinic-based education improves heart failure knowledge for patients with inadequate health literacy. However, further educational efforts for patients with inadequate health literacy are needed to reduce the disparity in knowledge between patients with inadequate and adequate health literacy.

Interestingly, patients with marginal health literacy did not improve over time. The relationship between marginal health literacy and heart failure knowledge is not a common focus of most research. Researchers in one study found no association between marginal health literacy and heart failure knowledge [17], although researchers in another study found that patients with marginal health literacy had significantly less knowledge than those with adequate health literacy [18]. Other researchers have taken the approach of collapsing the categories of inadequate and marginal health into one category of low health literacy. Further longitudinal research is needed to support our findings regarding marginal health literacy and heart failure knowledge.

We were surprised that over time, health literacy category was not associated with self-efficacy for heart failure self-care and self-care adherence in newly referred patients to a heart failure clinic. However, this could be due to a lack of power to detect differences. Posthoc power analyses revealed a lack of power in assessing self-efficacy (partial *η* ^2^ = 0.062, power = 0.277), self-care maintenance (partial *η* ^2^ = 0.065, power = 0.337), or self-care management (partial *η* ^2^ = 0.045, power = 0.089). Since the self-care management scale could only be scored if participants had symptoms in the prior two months, only 13 patients had scorable self-care management responses at all three assessments (participants with symptoms at baseline *N* = 39, two months *N* = 23, and four months *N* = 26) and were eligible for repeated measures ANOVA. 

In prior literature, relationships between health literacy and heart failure self-efficacy for self-care and self-care adherence were measured at only one point in time, and results were inconsistent. In a small, cross-sectional pilot study, researchers found no relationship between health literacy and self-efficacy [20], similar to our results. In larger studies, relationships between health literacy and self-efficacy differed from ours. When 95 patients with chronic heart failure were assessed during hospital admission, a significant relationship between health literacy and self-efficacy was found on univariate analysis, but the sample was too small to complete multivariate analysis [18]. It is unknown if whether self-efficacy or patient characteristics (age, gender, etc.) would be mediators for the relationship between health literacy and self-care had further analyses been performed. In a large sample (*N* = 605), self-efficacy was a mediator between health literacy and self-care in a structural equation model [19]. To our knowledge, our research provides the first examination of health literacy and self-efficacy longitudinally. Further research with larger sample sizes and adequately powered to detect differences is needed to examine these relationships over time. With larger samples, significant baseline factors can be controlled for to learn the importance of health literacy on outcomes. 

### 5.1. Limitations

Findings may be limited due to the majority of study participants having adequate health literacy scores. A new referral to a heart failure clinic may not necessarily mean a recent heart failure diagnosis. Patients may have had heart failure for some time and could have been treated elsewhere before referral. Previous heart failure education materials could have been developed based on low health literacy or reading levels, minimizing health literacy as an important factor in self-efficacy for self-care and self-care adherence. Prior education delivery and experiences in self-assessment and management of heart failure symptoms and outcomes of self-care behaviors could also have affected study findings, although heart failure knowledge, self-efficacy, and self-care adherence scores were below desired levels at baseline. 

Participant recruitment and retention may impact study findings and contributed to a lack of statistical power to assess self-efficacy and self-care. A total of 80 participants were initially enrolled, but 51 completed the study. An attempt was made to minimize attrition by making multiple attempts for followup at each assessment, and there were no significant differences in demographic characteristics between those at baseline and those who completed the study. We found significant associations between several demographic characteristics and study outcomes. In particular, younger participant age and more years of formal education were associated with higher heart failure knowledge. However, due to attrition, multivariate regression between participant characteristics and outcomes (heart failure knowledge, self-efficacy for self-care, and self-care adherence) or between recruitment site (taking into account educational or patient differences) and outcomes was unable to be performed. Future work should include these characteristics and should be adequately powered to better assess self-efficacy, self-care maintenance, and self-care management. 

Other limitations in this study include length of longitudinal assessment, potential of participants with mild cognitive dysfunction to be included, and the use of self-report measures that were valid and short but limited in scope. The four-month assessment (two months after education were completed) may not have been long enough to see the effects of health literacy on patient outcomes over time. However, Murray and colleagues [15] found that the effects of an educational intervention declined once the intervention ended, therefore, it is probable that the longitudinal effects could be seen at the four-month assessment. Future work should include a longer followup, such as six months or one year. While clinical judgment was utilized to exclude patients with cognitive impairment, some participants included in this study may have had undiagnosed mild cognitive impairment. Mild cognitive impairment has been found to lead to lower health literacy and poorer self-care and may have impacted results in this study.

## 6. Conclusions

Although health literacy was associated with patients' gain in heart failure knowledge over time, particularly in patients with low health literacy, health literacy was not associated with heart failure self-efficacy in performing self-care or self-care adherence. Examining the influence of health literacy on heart failure knowledge, self-efficacy for self-care, and self-care adherence over four months clarified some of the cross-sectional findings related to knowledge, self-efficacy, and self-care; however, these relationships are complex and merit further study. Investigators should examine approaches and work collaboratively with healthcare professionals to improve knowledge gains among inadequate health literacy patients during clinic-based education. 

## Figures and Tables

**Table 1:** Demographic information.

Demographic characteristic	All participants *N* = 51	Inadequate health literacy *N* = 10	Marginal health literacy *N* = 5	Adequate health literacy *N* = 36	*P* value
Age, mean (SD), y	64.68 (13.04)	77.00 (11.79)*	69.20 (10.76)	60.97 (11.71)*	0.002
Years of education, mean (SD), y	13.72 (2.77)	11.89 (2.67)	13.2 (1.79)	14.27 (2.75)	0.061
BMI, mean (SD), kg/m^2^	29.84 (8.14)	28.37 (5.59)	38.42 (13.74)*	29.06 (7.31)*	0.042
Prescription medications, mean (SD)	8.78 (4.28)	9.30 (3.53)	10.80 (1.79)	8.36 (4.66)	0.456
Recruitment site, *N* (%^a^)					0.018
Bloomington hospital	19 (37.3)	7 (13.7)	3 (5.9)	9 (17.6)	
Community health network	4 (7.8)	1 (2.0)	0 (0.0)	3 (5.9)	
Cleveland clinic	28 (54.9)	2 (3.9)	2 (3.9)	24 (41.1)	
Male, *N* (%)	29 (56.9)	5 (9.8)	3 (5.69)	21 (41.2)	0.885
Marital status, *N* (%)					0.025
Unmarried	5 (9.8)	1 (2.0)	1 (2.0)	3 (5.9)	
Married	34 (66.7)	3 (5.9)	2 (3.9)	29 (56.9)	
Divorced/separated	3 (5.9)	0 (0.0)	1 (2.0)	2 (3.9)	
Widowed	9 (17.6)	6 (11.8)	1 (2.0)	2 (3.9)	
Ethnicity, *N* (%)					0.287
Black/African American	3 (5.9)	0 (0.0)	1 (2.0)	1 (2.0)	
White/Caucasian	45 (88.2)	9 (17.6)	4 (7.8)	32 (62.7)	
Hispanic/Latino	3 (5.9)	1 (2.0)	0 (0.0)	1 (2.0)	
Financial status, *N* (%)					0.379
More than enough to make ends meet	22 (43.1)	4 (7.8)	1 (2.0)	17 (33.3)	
Enough to make ends meet	20 (39.2)	5 (9.8)	2 (3.9)	13 (25.5)	
Not enough to make ends meet	9 (7.6)	1 (2.0)	2 (3.9)	6 (11.8)	

*Significant difference between groups in posthoc tests, *P* < 0.05.

^a^All % calculated with a denominator of *N* = 51.

**Table 2:** Participant characteristics and their significant associations or differences in study outcomes.

	Knowledge			Self-efficacy		Self-Care maintenance
	Baseline	2 months	4 months	Baseline	2 months	4 months
Age*						
* r*	−0.342	−0.482	−0.339	—	—	—
*P*	0.015	<0.001	<0.001	—	—	—
Years of education*						
* r*	0.364	0.299	—	—	—	—
*P*	0.010	0.037	—	—	—	—
BMI*						
* r*	—	—	—	−0.339	−0.322	—
*P*	—	—	—	0.017	0.028	—
Recruitment site**						
*F*	6.535	—	—	4.425	—	3.824
*P*	0.003	—	—	0.017	—	0.029
Marital status**						
*F*	5.779	5.169	4.789	—	—	—
*P*	0.002	0.004	0.005	—	—	—

*Assessed using Pearson correlations.

**Assessed using One-Way Analysis of Variance.

**Table 3:** Health literacy, knowledge, self-efficacy, and self-care scores at baseline and followup overall and by health literacy level.

Group		Heart failure knowledge^a^		Self-efficacy^b^		Self-care maintenance^b^		Self-care management^b^	
	Assessment	Mean ± SD	Meaning	Mean ± SD	Meaning^c^	Mean ± SD	Meaning^c^	Mean ± SD	Meaning^c^
Overall	Baseline	8.2 ± 2.7	54.7% correct	69.6 ± 19.4	Not adequate	69.5 ± 16.9	Not adequate	64.3 ± 21.5	Not adequate
	2 months	9.3 ± 3.3	62.0% correct	72.2 ± 15.5	Adequate	76.3 ± 14.9	Adequate	73.4 ± 18.5	Adequate
	4 months	9.6 ± 2.4	64.0% correct	75.0 ± 16.0	Adequate	76.3 ± 14.5	Adequate	70.6 ± 19.7	Adequate

Inadequate health literacy	Baseline	5.3 ± 2.4	35.3% correct	64.2 ± 21.9	Not adequate	63.9 ± 21.7	Not adequate	52.9 ± 32.4	Not adequate
	2 months	5.9 ± 2.5	39.3% correct	72.3 ± 16.0	Adequate	69.7 ± 17.9	Not adequate	68.0 ± 20.2	Not adequate
	4 months	7.8 ± 1.7	52.0% correct	82.8 ± 19.4	Adequate	68.9 ± 15.2	Not adequate	68.6 ± 31.5	Not adequate

Marginal health literacy	Baseline	9.0 ± 1.6	60.0% correct	54.5 ± 10.0	Not adequate	63.3 ± 18.6	Not adequate	70.0 ± 22.0	Adequate
	2 months	9.0 ± 2.9	60.0% correct	67.8 ± 10.7	Not adequate	80.0 ± 13.3	Adequate	65.0 ± 35.4	Not adequate
	4 months	8.8 ± 3.8	58.7% correct	66.8 ± 6.7	Not adequate	76.7 ± 10.5	Adequate	62.5 ± 24.7	Not adequate

Adequate health literacy	Baseline	8.8 ± 2.3	58.7% correct	73.0 ± 18.8	Adequate	72.0 ± 15.1	Adequate	66.5 ± 17.8	Not adequate
	2 months	10.2 ± 3.0	68.0% correct	76.3 ± 16.2	Adequate	77.6 ± 14.1	Adequate	76.3 ± 16.6	Adequate
	4 months	10.3 ± 2.1	68.7% correct	74.3 ± 15.8	Adequate	76.3 ± 14.5	Adequate	72.4 ± 13.4	Adequate

^a^Possible range 0–15.

^b^Possible range 0–100.

^c^Adequacy, according to the SCHFI, is at scores ≥70.

**Table 4:** Longitudinal effects of health literacy on outcomes using repeated measures analysis of variance.

Effect	Knowledge		Self-efficacy		Self-care maintenance		Self-care management	
	*F*	*P*	*F*	*P*	*F*	*P*	*F*	*P*
Time	3.519	0.034	1.954	0.148	6.942	0.002	0.285	0.754
Health literacy	11.096	<0.001	1.364	0.267	1.682	0.197	0.307	0.741
Time∗health literacy	1.189	0.131	1.037	0.393	0.707	0.589	1.376	0.269
